# Glacier advance during Marine Isotope Stage 11 in the McMurdo Dry Valleys of Antarctica

**DOI:** 10.1038/srep41433

**Published:** 2017-01-31

**Authors:** Kate M. Swanger, Jennifer L. Lamp, Gisela Winckler, Joerg M. Schaefer, David R. Marchant

**Affiliations:** 1 Department of Environmental, Earth and Atmospheric Sciences, University of Massachusetts, 1 University Avenue, Lowell, MA 01854, USA; 2 Lamont-Doherty Earth Observatory, The Earth Institute at Columbia University, Route 9W, Palisades, NY 10964, USA; 3 Department of Earth and Environment, Boston University, 685 Commonwealth Avenue, Boston, MA 02215, USA

## Abstract

We mapped six distinct glacial moraines alongside Stocking Glacier in the McMurdo Dry Valleys, Antarctica. Stocking Glacier is one of several alpine glaciers in the Dry Valleys fringed by multiple cold-based drop moraines. To determine the age of the outermost moraine, we collected 10 boulders of Ferrar Dolerite along the crest of the moraine and analyzed mineral separates of pyroxene for cosmogenic ^3^He. On the basis of these measurements, the exposure age for the outermost moraine is 391 ± 35 ka. This represents the first documented advance of alpine glacier ice in the Dry Valleys during Marine Isotope Stage (MIS) 11. At this time, Stocking Glacier was ~20–30% larger than today. The cause of ice expansion is uncertain, but most likely it is related to increased atmospheric temperature and precipitation, associated with reduced ice extent in the nearby Ross Embayment. The data suggest complex local environmental response to warm climates in Antarctica and have implications for glacial response to Holocene warming. The study also demonstrates the potential for using alpine glacier chronologies in the Transantarctic Mountains as proxies for retreat of grounded glacier ice in the Ross Embayment.

The McMurdo Dry Valleys contain one of the longest terrestrial records of glaciation in Antarctica, extending from the mid-Miocene to the present^1^,^2^. Nestled between the Polar Plateau and the Ross Sea, the record of glaciation in the Dry Valleys reflects changes in the volume of East Antarctic ice as well as the extent of open-water and grounded-glacier ice in the Ross Embayment (Fig. 1)^3^,^4^.

Here we present an alpine exposure-age chronology from the Dry Valleys. This chronology comes from Stocking Glacier, which is situated in the central Transantarctic Mountains at ~77°42′S and 161°51′E, near the center of the Dry Valleys region. The glacier descends from three cirques in the Asgard Range and terminates in central Taylor Valley, ~350 m above the margin of Taylor Glacier, an outlet glacier of the East Antarctic Ice Sheet. Taylor Valley is ~50-km long, 7- to 10-km wide, and home to dozens of alpine glaciers that flow downslope from the Asgard Range in the north and the Kukri Hills in the south (Fig. 1).

The Dry Valleys are classified as a cold-polar desert, with a mean annual air temperature of –20 °C and snowfall precipitation of 20–300 mm of water equivalent per year^5^,^6^. In detail, both temperature and precipitation vary markedly across the region, with summertime (DJF) air temperatures near the coast rising several degrees above 0 °C for more than a month, even though temperatures in the high-elevation interior persist below 0 °C during the summertime^7^. Precipitation is generally greatest near the coast^6^. As defined in ref. 7 Stocking Glacier is located at the geographic transition between the relatively warm coastal-thaw zone and the inland-mixed zone, where modern summertime air temperatures occasionally rise above 0 °C for a few weeks per year.

Although annual and summertime ice loss at Stocking Glacier is dominated by sublimation, local meltwater forms alongside solar-heated rocks exposed on the glacier surface^8^, and more significantly at lateral-ice margins where supraglacial debris is concentrated (Figs 1 and 2). Like the other alpine glaciers in the Dry Valleys region, Stocking Glacier is fed from local snowfall. The majority of ice accumulation for Stocking Glacier occurs in the westernmost of the three source cirque valleys in the Asgard Range (Fig. 1). From these local accumulation areas, Stocking Glacier flows for ~6 km, descending from ~1800 meters above sea level (masl) to ~800 masl. Presently, Stocking Glacier covers 9.0 km^2^. Based on glacier geometry and the contour inflection method^9^, we infer that the modern equilibrium line altitude (ELA) for Stocking Glacier is ~1300 ± 50 masl, in agreement with earlier estimates^10^. We note, however, that alpine glaciers in the Dry Valleys do not have traditional and easily mapped ice-accumulation and ice-ablation zones. This is because sublimation dominates, and accumulation-ablation dynamics are impacted to a great extent by local and variable winds, which give rise to patchworks of blue-ice ablation areas and intervening accumulation areas^11^. As such, equilibrium lines are complex and irregular.

Given the low atmospheric temperatures, the surrounding topography, and flow data from similar alpine glaciers in Taylor Valley, ice-flow velocities for Stocking Glacier are likely <5 m/yr^10^. Consequently, Stocking Glacier is almost certainly frozen to its bed, and thus rates of basal erosion are negligible over the timescales considered here^12^,^13^. Like other glaciers in Taylor Valley^11^,^14^, the terminus of Stocking Glacier is an ice cliff, 20–30 m high, that calves onto a low-angle ice apron below (Figs 2 and 3). This ice apron is a composite of ice chunks, occasional boulders, fine sediments, and refrozen meltwater. Presently, there is no moraine forming at the terminus. The lateral margins in the ablation zone of Stocking Glacier are characterized by stepped calving, seasonally-active meltwater streams, and ice-cored moraines overlain by fluvially-reworked, sand-rich sediments^15^.

Like other alpine glaciers in Taylor Valley, Stocking Glacier lacks significant basal and supraglacial debris, as well as associated matrix-supported moraines. Instead, former ice-marginal positions for Stocking Glacier are marked by discontinuous lines of isolated boulders and cobbles. These aligned clasts have been termed boulder-belt moraines^16^ and drop moraines^7^,^13^, and originate as rocks fall sporadically from cirque headwalls onto the glacier surface. Following initial emplacement, these clasts are transported via englacial and/or supraglacial pathways, and are deposited at the margin of terminal ice cliffs (Figs 2 and 3).

## Results

### Stocking Glacier drop moraines and ice-cored drift

**Figure 1:**
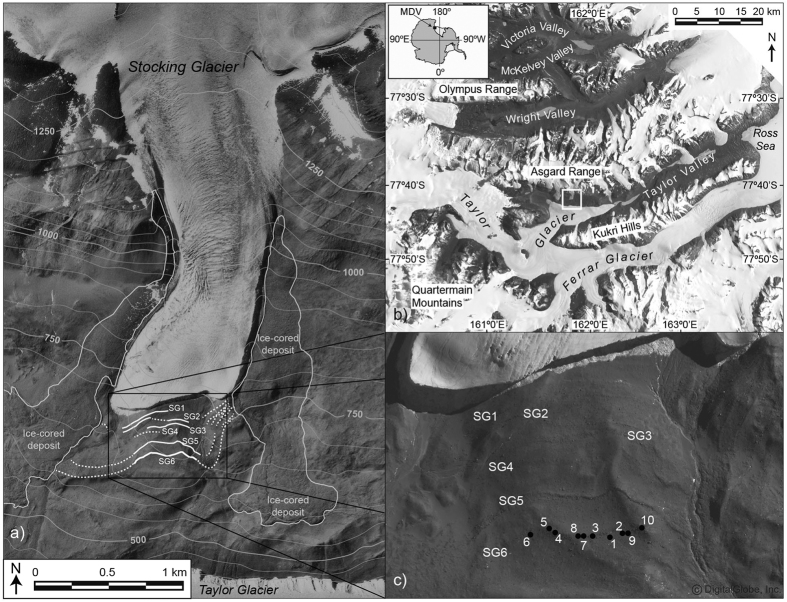
(**a**) Stocking Glacier and surrounding region on the north wall of Taylor Valley. Drop moraines are traced and labeled in white. Ice-cored lateral deposits are outlines in grey. Southern lateral deposits show geomorphic evidence of flow, but northern deposits (adjacent to present glacier) do not. (**b**) McMurdo Dry Valleys (MDV) in the Transantarctic Mountains with inset of Antarctica showing location of Dry Valleys. White box indicates location of Stocking Glacier. (**c**) Close-up view of Stocking Glacier moraines with exposure sample locations; numbers correspond to sample number (1 is DVX-11-01, 2 is DVX-11-02, etc.). Image/data sources: (**a**) public domain aerial photograph from U.S. Geological Survey, TMA2480-V0120 taken in 1983, courtesy of the Polar Geospatial Center. Topographic contours are from the public domain Lake Vanda Quadrangle (1977, 1:50,000 Topographic Series, U.S. Geological Survey, Reston, VA), rectified with the aerial photograph in ArcMap 10, www.esri.com. (**b**) Public domain Landsat7 imagery courtesy of NASA Goddard Space Flight Center and U.S. Geological Survey. (**c**) This figure is not covered by the CC BY license. GeoEye-1 Satellite image ©2013 DigitalGlobe. All Rights Reserved. Courtesy of the Polar Geospatial Center. Used with permission. All manuscript figures were created in Adobe Illustrator CC 2015, www.adobe.com/illustrator.

**Figure 2:**
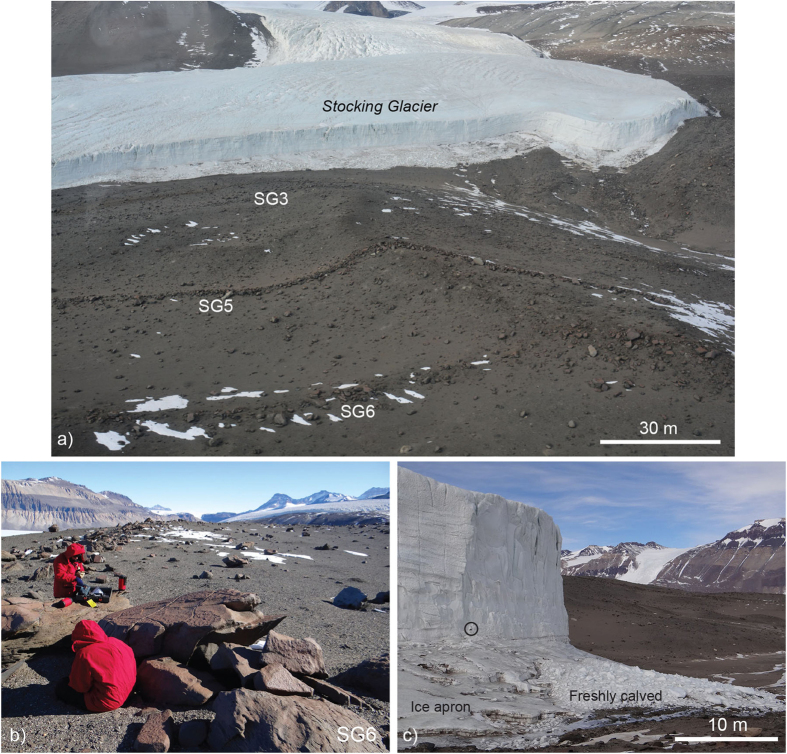
(**a**) Oblique aerial photograph of Stocking Glacier margin. Note ice cliff, ice apron and the lack of significant supraglacial, englacial, or basal debris. Moraines SG3, SG5 and SG6 (the outermost moraine) are visible. (**b**) Ground photograph of the outermost moraine (SG6), looking west toward Taylor Dome; note small-scale pitting, wind faceting and oxidation of boulder surfaces. (**c**) Close-up view of the terminus of modern Stocking Glacier, showing ice cliff, ice apron and freshly calved ice blocks. Black circle shows an entrained clast. Melting along solar heated rocks and sand grains is sufficient to produce small meltwater streams that drain southward toward Taylor Glacier (see also Fig. 1). All images courtesy of K.M. Swanger, reprinted with permission.

Deposits from Stocking Glacier fall into two general categories: 1) the boulder-rich drop moraines and 2) ice-cored drift. The drop moraines occur solely beyond the glacier terminus, whereas ice-cored drifts dominate lateral ice margins. The latter are associated with small meltwater streams that deposit sands and gravels^15^ and morphologically cover >1 km^2^ (Figs 1 and 2). The distribution of these two distinct drifts is in accordance with present-day depositional processes alongside Stocking Glacier, with the implication being that similar ice-cliffs, ice aprons and meltwater dynamics operated during past advances of Stocking Glacier (Fig. 2).

Six drop moraines lie distal and subparallel to the modern terminus of Stocking Glacier. The moraines rest on a bedrock bench that dips away from the glacier snout at ~15°. Moraines are designated SG1 through SG6, with SG1 closest to the modern glacier margin (Fig. 1). The outer two moraines (SG5 and SG6) are the best developed of the sequence and in places exhibit boulder stacking and well-formed ridges. Both SG5 and SG6 extend continuously for ~1 km in front of the glacier, but become diffuse and intermittent as they bend toward lateral ice-rich deposits. Although the precise geometry of each moraine varies along trace, the average heights for SG5 and SG6 are 1–3 m, and widths are 6–12 m (Fig. 2). Moraines SG1 through SG4 are shorter (~200–400 m in length) and more diffuse, with scattered boulders often spaced > 2 m apart.

**Figure 3:**
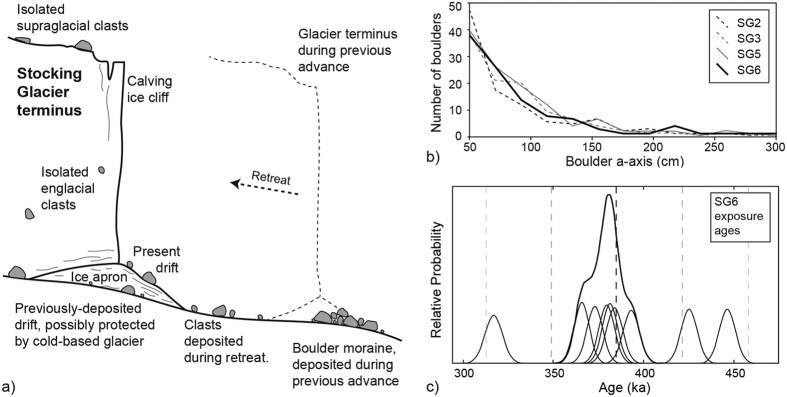
(**a**) Schematic of depositional processes near the terminus of Stocking Glacier (for other examples of cold-based glaciers from the Dry Valleys and the importance of ice aprons in depositional processes and glacier dynamics, please see refs 38, 39, 40). Stocking Glacier is cold-based and frozen to the substrate across its entire length. (**b**) Clast size frequency plot for boulders (>40 cm in width) from moraines SG2, 3, 5 and 6 (SG4 lacked sufficient boulders for analysis). Data are binned into 20 cm intervals (40–60 cm, 60–80 cm, etc.). The similar clast size distribution is indicative of limited post-depositional rock breakdown across the moraine sequence. (**c**) Exposure age probability density function plot for nine samples from SG6. All nine samples yield a weighted mean exposure age of 391 ± 35 ka, assuming no erosion.

All six moraines (SG1–SG6) are homogeneous in terms of lithology, average clast-size distribution, surface characteristics, and weathering state (Figs 2 and 3). Moraines are composed predominantly of Ferrar Dolerite. Clasts lack evidence of transport by wet-based ice (there are no striations or polished surfaces), and instead exhibit minor faceting by wind erosion and sand blasting; most retain angular features inherited during rock-fall deposition onto the glacier surface. Nearly all boulders exhibit minor surface oxidation, though there is no observable variation in these parameters across the moraine set. From SG1 to SG6, all appear equally weathered. Based on Schmidt Hammer tests, there is a slight decrease in the compressional strength of rock surfaces with increasing distance from the modern glacier. However, compressional rock strength measurements across all sampled boulders from SG2 to SG6 are within error of each other, implying minimal chemical alteration of boulders. These shared characteristics suggest similar source areas and entrainment processes for each clast. The similar boulder-size distribution and surface weathering characteristics across all deposits also suggests that the moraines have experienced minimal rock erosion. The latter assertion is consistent with calculated erosion rates for the Dry Valleys, which range from 5–20 cm/Myr in upland moraines and bedrock surfaces^17^,^18^,^19^.

### Stocking Glacier during maximum recorded ice advance

**Figure 4:**
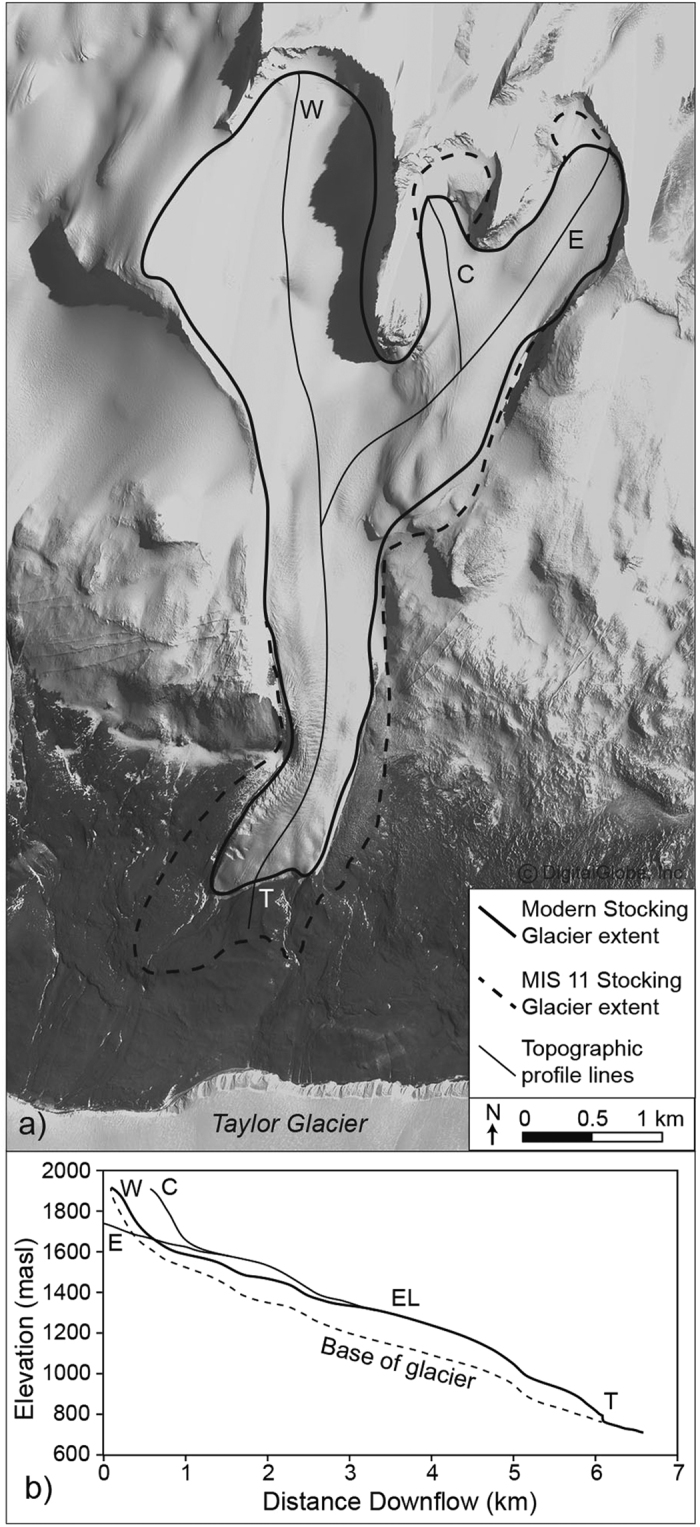
(**a**) Satellite image of Stocking Glacier. The modern glacier is outlined with solid line, including three distinct source areas, west (W), central (C), and east (E). The mapped extent of Stocking Glacier during its greatest recorded advance (SG6) is outlined with dashed lines. (**b**) Topographic profile of Stocking Glacier. Glacier thickness estimates (shown by the dashed lines) are largely inferred and bracketed between 50–200 m, the average glacier thicknesses for Dry Valley alpine glaciers^10^. This image is not covered by the CC BY license. GeoEye-1 Satellite image ©2013 DigitalGlobe.

To determine the areal distribution of Stocking Glacier during its maximum-recorded ice advance, we integrated the position of the outermost drop moraine, SG6, with the maximum extent of the lateral, ice-cored drift. However, some of the ice-cored drift alongside Stocking Glacier has advanced downslope after initial deposition due to ice creep^15^, and thus cannot be used for maximum ice reconstructions. Southern (distal) sections, for example, display compressional flow features, such as transverse steps and risers, whereas the ice-cored drift in the north lacks such features and is instead characterized by symmetrical contraction-crack polygons, both suggestive of minimal downslope displacement^20^. Therefore, when mapping maximum ice extent, we only include the northern portion of ice-cored drift, and exclude southern portions beyond the glacier terminus that show signs of flow (Figs 1 and 4). Given this, we find that during maximum ice extent (from SG6 and northern lateral drifts), the terminus of Stocking Glacier extended 450–500 m beyond its present margin and lateral margins extended ~200 m. At this time, Stocking Glacier achieved a total ice surface area of 11.0–11.5 km^2^, resulting in an increase in the areal extent of Stocking Glacier by 20–30% over present values (Fig. 4).

### Exposure-age chronology

A depositional age for the outermost moraine from Stocking Glacier comes from cosmogenic ^3^He measured in pyroxene crystals from 10 surface boulders of Ferrar Dolerite from the surface of SG6 (Table 1). Except for one clast, DVX-11-05, which yielded an exposure age of 915 ka, all measured clasts on SG6 showed strong age agreement, indicating uniform entrainment and depositional processes. The outlier, at 915 ka, likely contains inherited ^3^He from a prior exposure. Excluding this sample, the nine remaining samples yield a weighted mean exposure age of 391 ± 35 ka (Fig. 3). Applying a constant erosion rate of 10 cm/Myr^17^,^18^,^19^,^21^, the modeled exposure age for SG6 is ~400–405 ka. Of the nine samples, the exposure ages of six samples fall within 1σ (Fig. 3). The weighted mean exposure age for these six samples is 382 ± 10 ka. Notably, the calculated moraine age does not change significantly based on whether we include all nine samples or only the six clustered samples. Given the above, our results suggest that the outmost moraine from Stocking Glacier (SG6) dates to Marine Isotope Stage (MIS) 11. The age range for MIS 11, as recorded in deep-sea cores, extends from ~424 to ~374 ka^22^.

Because cold-based glaciers lack significant erosive potential^23^, Stocking Glacier could have advanced repeatedly over previously-deposited moraines without substantial reworking. In this scenario, the potential for burial beneath advancing ice, and possible superposed deposition of isolated clasts, would be greatest for moraines inboard of maximum ice advance (e.g., SG6). If correct, then moraines SG1–SG5 could have been overrun at multiple times, precluding straightforward dating of the moraines via cosmogenic-nuclide analyses of surface boulders. Additional analyses are required to test this assertion and to help define depositional ages for these moraines.

## Discussion

The Stocking Glacier terminal moraine (SG6) is the first alpine moraine in the Dry Valleys dated to MIS 11. Previously, U/Th dates on carbonates deposited in proglacial lakes in Taylor Valley, as well as cosmogenic-nuclide exposure dating of moraines in the Quartermain Mountains, show advance of Taylor Glacier during MIS 5, 7, 9 and possibly 11^19^,^24^,^25^. The new alpine chronology presented here, when combined with these previous datasets, suggests that both outlet and alpine glaciers in the Dry Valleys were larger than present during MIS 11. It is important to note that during deposition of SG6, Stocking Glacier did not merge with (and flow into) Taylor Glacier, thus providing constraints on the extent of both Stocking Glacier and Taylor Glacier at this time.

Cold-based alpine glaciers in the Dry Valleys display complex response to climate change, primarily because mean annual air temperatures are exceedingly low, –20 °C. The driving forces for advance and retreat are not straightforward and thus can be quite different from temperate alpine glaciers^26^. For example, Dry Valley alpine glaciers are more extensive now than during the worldwide Last Glacial Maximum (LGM)^11^ at ~18 ka. This finding, along with other data^3^,^24^, prompted many to suggest that alpine glaciers in the Dry Valleys advance during warm intervals and retreat during cold intervals. Our data that call for expanded alpine ice during MIS 11 are consistent with this assertion. The key remaining questions are: What specific factors lead to ice advance during warm intervals? And why was advance during MIS 11 greater than during the Holocene?

In general, glacial advance during warm intervals in the Dry Valleys is most likely caused by a) increased precipitation, b) weaker ice rheology, c) increased cloudiness, d) lower mean wind velocities, and/or e) higher albedo ice surfaces^14^,^26^,^27^.

In the Dry Valleys, modest atmospheric warming of a few degrees Celsius (without an accompanying increase in precipitation) weakens ice rheology and permits terminal advance through flow-induced thinning^27^. However, based on the models and observations presented in ref. 27, small alpine glaciers, like Stocking Glacier, experience relatively minor ice-front advance due to weaker rheology. For example, a ~2 °C increase in atmospheric/ice temperature would yield an ice-front advance of ~25 m^27^, well short of the ~500 m of advance associated with the SG6 moraine.

Modern precipitation in the Dry Valleys is locally sourced from open water in the nearby Ross Sea^28^. During glacial intervals, however, grounded ice and floating ice shelves in the Ross Sea advanced north toward the edge of the continental shelf. The expansion increased the distance to open water from <100 km to ~1,000 km, effectively isolating the Dry Valleys from their primary moisture source^29^,^30^. Consequently, precipitation rates in the Dry Valleys are reduced during glacial intervals and enhanced during interglacial intervals. Further, ice accumulation data from Taylor Dome, situated ~50 km southwest of Stocking Glacier, suggest a six-fold increase in precipitation rates during the Holocene relative to the LGM^31^.

Winds and albedo directly impact ablation and ice fluctuations. In the Dry Valleys, ablation is dominated by sublimation year-round, with some near surface (0–50 cm depth) melt during the summer months^14^,^26^. Sublimation rates increase significantly during katabatic wind storms, and in general increased winds lead to increased ablation^14^. Near-surface melting increases with decreasing ice-albedo, especially areas with blue ice, fine sediment layers, or entrained clasts^8^,^14^. It is therefore possible that ice-margin retreat during glacial maxima may have been exacerbated by increased ablation from enhanced winds and airborne dust^29^.

Cloudiness may increase or decrease ablation and therefore has a multi-directional control on glacial dynamics. As expected, increased cloudiness during the summer can result in lower sublimation rates by decreasing shortwave radiation and increasing atmospheric humidity^26^,^31^. However, increased cloudiness can also cause increased melting as a result of the “radiation paradox” in which cloud cover results in a gain in longwave radiation that overcompensates for any loss in shortwave radiation^14^.

Our data suggest that during MIS 11, Stocking Glacier advanced 450–500 m compared to the present, with an increase in areal extent of 20–30%. This advance is too great to be explained by weaker ice rheology alone and therefore signifies a secondary response tied to increased accumulation and/or decreased ablation. Given the noted controls on ice dynamics discussed above, we follow^10^,^24^ and favor increased precipitation, caused by a reduction in sea ice, floating ice shelves, and grounded-glacier ice in the Ross Embayment, as the most likely dominant cause of glacier advance during MIS 11.

The AND 1-B marine sediment core provides a high-resolution record of glacial advance and retreat in the Ross Embayment from the Pliocene to the present (located 100 km offshore of the Taylor Valley coast)^32^. Based on the AND-1B sediment record, the Ross Ice Shelf (RIS) has experienced substantial retreat during MIS 31–33 (~1.1 Ma)^32^,^33^. Following this period, however, sediments in the AND-1B core dominantly reflect glacial advance (diamict), except for a few recent interglacial intervals, characterized by deposition of fine sediment and diatoms^32^.

Of the recent interglacial intervals, MIS 11 experienced similar insolation-forcing as the Holocene^34^,^35^,^36^. Peak warmth during MIS 11 was slightly cooler than during MIS 5, but lasted considerably longer than other recent interglacials^37^. Sediments recovered from the AND-1B core, along with results from coupled climate-ice sheet models, suggest that ice-volume estimates in the Ross Sea during MIS 11 were similar to other late-Pleistocene interglacial intervals^32^,^33^. If correct, then the anomalous length of MIS 11, rather than excessive atmospheric warming or extreme ice recession in the Ross Embayment, might have been the key factor in dictating the extent of alpine glacier advance in the Dry Valleys. The dated terminal moraine, SG6, highlights the complexity of Dry Valley alpine glacier response to climate fluctuations, especially the potential importance of the magnitude of warming vs. the duration of warming.

## Methods

### Field mapping, sedimentology and weathering

We employed geomorphic analyses of orthorectified aerial photographs and detailed fieldwork in 2011 and 2013 to map drifts deposited from Stocking Glacier. Elevation control was established using hand-held GPS units, a Garmin 3000 with a reported accuracy of ± 5 m horizontal and ± 10 m vertical and a Trimble GeoExplorer 6000 with ± 1 m horizontal/vertical accuracy. Drift characteristics for drop moraines were quantified through detailed tape-and-compass mapping of moraine height and width (Supplementary Figures and Tables). Clast dimensions were measured for 100 boulders from each of the moraines (SG2, 3, 5, and 6) using a Zingg classification system; only clasts >40 cm (a-axis) were measured. During the course of fieldwork we collected sediment samples at 20- to 30-cm depth intervals (each 2 kg) for standard grain-size analyses. We examined the 16- to 64-mm fraction (gravel) for lithologic constituents and evidence for surface modification (such as glacial scour and/or weathering). Weathering processes and rates were measured through qualitative documentation of the presence of oxidation rinds and salt pits and quantitative measure of rock strength (a relative chronology of exposure to weathering processes). We used a Type N Schmidt Hammer to determine rebound (R) values on 50 clasts on each moraine (except SG4, which lacked sufficient boulders). Each clast was measured three times. Higher R-values indicate stronger, less weathered clasts (Supplementary Figures).

### Equilibrium line mapping

The present equilibrium line for Stocking Glacier was calculated using a contour inflection method^8^. The ELA is inferred to coincide with the point on the glacier in which it transitions from concave outward (accumulation zone) to convex outward (ablation zone).

### Cosmogenic ^3^He sample collection

We collected surface clasts (Ferrar Dolerite) from the six mapped drop moraines and the right lateral ice-cored moraine for cosmogenic-nuclide analyses. To minimize the effects of potential rock displacement associated with the development of contraction-crack polygons, we restricted sample collection to areas without polygons. We also collected samples along ridge crests, in order to reduce the chances of burial beneath wind-blown snow. In the field, for each sample we measured a) topographic shielding, b) the strike and dip of the rock surface, c) the boulder size, d) the latitude and longitude, and e) the elevation. Samples were removed from the surface of the boulders using a wedge and shim technique.

### Mineral separation and gas extraction

The upper 2–4 cm of the samples were cut and crushed at the University of Massachusetts using a Bico Pulverizer; fragments were then sieved to isolate the >150 μm and <500 μm fraction. Typical sample weights were about 300 g. Additional physical and chemical processing was performed in the cosmogenic laboratory at Lamont-Doherty Earth Observatory (LDEO). Samples were further crushed to 63–125 μm to produce separate mineral grains, rinsed thoroughly with deionized (DI) water to remove fines, then leached in a 10% phosphoric acid solution to remove oxidation and other contaminants from mineral surfaces. The samples were then density-separated in a centrifuge using sodium polytungstate heavy liquid at a density of 3.0 g/cm^3^; the sinking fraction was rinsed with DI water and isopropanol, and allowed to dry overnight. Magnetic minerals were removed using a Frantz magnetic separator set between 0.2 and 0.4 amps. The non-magnetic fraction was then leached in a 2% HNO_3_ / 2% HF solution on a shaker table overnight (~15–20 hours), then rinsed, dried, and packed in aluminum foil for analysis. Helium isotopes were measured with the LDEO MAP 215–50 noble gas mass spectrometer calibrated with a known volume of a Yellowstone helium standard (Murdering Mudpots) with a ^3^He/^4^He ratio of 16.45*R*_*a*_, where *R*_*a*_ = (^3^He/^4^He)_air_ = 1.384 × 10^−6^. Hot procedural blanks contained less than 10^−15^ cc STP of ^3^He and less than 10^−9^ cc STP of ^4^He with approximately atmospheric helium isotopic composition. Blank corrections for ^3^He were smaller than 1% for all samples.

## Additional Information

**How to cite this article**: Swanger, K. M. *et al*. Glacier advance during Marine Isotope Stage 11 in the McMurdo Dry Valleys of Antarctica. *Sci. Rep.* **7**, 41433; doi: 10.1038/srep41433 (2017).

**Publisher's note:** Springer Nature remains neutral with regard to jurisdictional claims in published maps and institutional affiliations.

## Supplementary Material

Supplementary Figure and Table

## Figures and Tables

**Table 1:** Cosmogenic ^3^He exposure ages for Stocking Glacier outer moraine.

Sample	Altitude^a^ (masl)	Latitude^a^ (S)	Longitude^a^ (E)	Sample thickness^b^ (cm)	Shielding correction^c^	^3^He (10^8^ at/g)^d^	^3^He exposure age (ka)^e^	Exposure age 10 cm/Myr erosion (ka)^f^
DVX-11-01	697	77°43′06″	161°50′56″	4	0.994	1.208 ± 0.012	426 ± 4	443
DVX-11-02	697	77°43′06″	161°50′58″	3	0.995	1.279 ± 0.013	447 ± 4	466
DVX-11-03	699	77°43′06″	161°50′49″	3	0.995	1.073 ± 0.012	376 ± 4	389
DVX-11-04	697	77°43′06″	161°50′42″	2	0.997	0.926 ± 0.014	322 ± 5	331
DVX-11-05	693	77°43′06″	161°50′42″	5	0.991	2.564 ± 0.021	915 ± 8	997
DVX-11-06	694	77°43′06″	161°50′39″	3	0.997	1.095 ± 0.011	384 ± 4	398
DVX-11-07	705	77°43′06″	161°50′48″	3	0.995	1.096 ± 0.012	382 ± 4	396
DVX-11-08	698	77°43′06″	161°50′49″	3	0.991	1.124 ± 0.013	396 ± 5	410
DVX-11-09	703	77°43′06″	161°50′59″	3	0.987	1.103 ± 0.012	387 ± 4	400
DVX-11-10	703	77°43′06″	161°51′03″	3	0.988	1.050 ± 0.011	369 ± 4	382

^a^Longitude, latitude and altitude (masl = meters above sea level) were measured at each sample location using a Trimble GeoExplorer 6000.

^b^We used a sea level, high-latitude cosmogenic ^3^He production rate of 120 at g^−1^ yr^−1^ (pyroxene)^41^. Cosmogenic production rates were scaled for elevation using equations for Antarctica^42^. Attenuation of production with depth was calculated assuming an attenuation length of 160 g cm^−2^ and an average rock density of 2.8 g cm^−3^.

^c^Shielding factors were calculated from horizon geometry measurements recorded for each sample in the field^43^.

^d^1σ errors of ^3^He concentrations reflect propagated analytical uncertainties, based on statistical errors and variability in the sensitivity of the mass spectrometer.

^e^Minimum ages assume no erosion, accounting only for production rates, sample thickness, and shielding factors at each sample location.

^f^Exposure ages assuming a constant erosion rate of 10 cm/Myr.
