# Supplementary material for: Glacier advance during Marine Isotope Stage 11 in the McMurdo Dry Valleys of Antarctica
Source: Sci Rep. 2017 Jan 31;7:41433. doi: 10.1038/srep41433 (PMC5282522; doi:10.1038/srep41433)
Supplement: Supplementary Figure and Table [file srep41433-s1.pdf]

Supplementary Information

Glacier advance during Marine Isotope Stage 11 in the McMurdo Dry Valleys of Antarctica

Kate M. Swanger[1,*], Jennifer L. Lamp[2,3], Gisela Winckler[2], Joerg M. Schaefer[2] and David R. Marchant[3]

[1] Department of Environmental, Earth and Atmospheric Sciences, University of Massachusetts, 1 University Avenue, Lowell, MA 01854, USA

[2] Lamont-Doherty Earth Observatory, The Earth Institute at Columbia University, Route 9W, Palisades, NY 10964, USA

[3] Department of Earth and Environment, Boston University, 675 Commonwealth Avenue, Boston, MA 02215, USA

* Corresponding author. Kate_Swanger@uml.edu

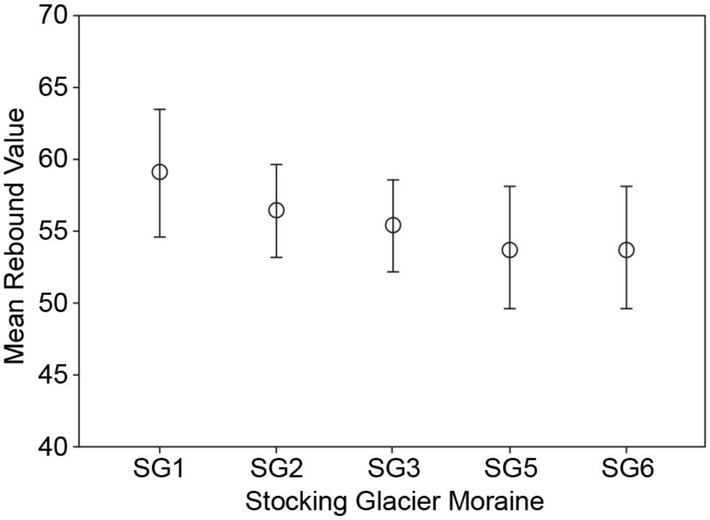

Fig. S1. In order to understand more about the internal weathering of clasts at our study site, we used a Type N Schmidt Hammer to determine rebound (R) values for 50 clasts on each moraine of the study sites (three rebound measurements per sample). Higher R-values indicate stronger, less weathered clasts, and smaller R-values indicate weaker rocks. At Stocking Glacier there is a general trend in decreasing R-value of dolerites with increasing moraine age, indicating slightly more weathered dolerites in moraines exposed the longest. However, all Schmidt Hammer data fall within error and therefore the decrease in rock strength is minimal and inconclusive. The Schmidt Hammer measures the distance of rebound of a controlled impact on a rock surface. The Type N Schmidt Hammer can measure compressive strengths from 20–250 MPa.

Supplementary Table 1. Dimensions and trace for Stocking Glacier Moraine SG6.

Moraine	Latitude[1]	Longitude[1]	Elevation (masl)[1]	Moraine width (m)[2]
SG6	77º43'05.7"	161º50'37.2"	690	9.6
	77º43'05.8"	161º50'40.7"	692	15.6
	77º43'06.1"	161º50'44.1"	693	12.3
	77º43'06.1"	161º50'47.4"	693	6.9
	77º43'06.2"	161º50'51.3"	696	6.1
	77º43'06.1"	161º50'54.6"	695	10.2
	77º43'05.9"	161º50'58.3"	695	8.3
	77º43'05.6"	161º51'01.8"	698	8.9
	77º43'05.5"	161º51'05.3"	700	8.9
	77º43'05.3"	161º51'08.5"	703	21.4
	77º43'05.8"	161º51'12.0"	703	21.6
	77º43'06.1"	161º51'15.1"	698	22.3
	77º43'06.8"	161º51'19.8"	695	10.5
	77º43'07.3"	161º51'22.0"	692	7.4

[1] Latitude, longitude and elevation were measured in the field using a Trimble GeoExplorer 6000. Horizontal error is 5–6 m. Vertical error is 15–20 m.

[2] Moraine width was measured perpendicular to the moraine trace, based on the visual extent of concentrated boulder scatter.
